# Role of volcanism and impact heating in mass extinction climate shifts

**DOI:** 10.1038/s41598-024-60467-y

**Published:** 2024-04-30

**Authors:** Kunio Kaiho

**Affiliations:** Department of Earth Science, Graduate School of Science, Tohoku University, Sendai, 980-8578 Japan

**Keywords:** Volcanic activity, Asteroid impact, Heating temperature, Emitted gases, Climate variations, Mass extinction, Climate sciences, Ecology, Environmental sciences, Planetary science, Solid Earth sciences

## Abstract

This study investigates the mechanisms underlying the varied climate changes witnessed during mass extinctions in the Phanerozoic Eon. Climate shifts during mass extinctions have manifested as either predominant global cooling or predominant warming, yet the causes behind these occurrences remain unclear. We emphasize the significance of sedimentary rock temperature in comprehending these climate shifts. Our research reveals that low-temperature heating of sulfide leads to global cooling through the release of sulfur dioxide (SO_2_), while intermediate-temperature heating of hydrocarbons and carbonates releases substantial carbon dioxide (CO_2_), contributing to global warming. High-temperature heating additionally generates SO_2_ from sulfate, further contributing to global cooling. Different degrees of contact heating of the host rock can lead to different dominant volatile gas emissions, crucially driving either warming or cooling. Moreover, medium to high-temperature shock-heating resulting from asteroid impacts produces soot from hydrocarbons, also contributing to global cooling. Large-scale volcanic activity and asteroid impacts are both events that heat rocks, emitting the same gases and particles, causing climate changes. The findings elucidate the critical role of heating temperature and heating time in understanding major climate changes during mass extinctions.

## Introduction

While numerous studies have sought to establish broad generalizations concerning the causal relationship between the emplacement of Large Igneous Provinces (LIPs) or bolide impacts and global environmental changes, limited attention has been given to exploring the intricate details of this connection^1–4^. This paper aims to address this gap by presenting experimental data that indicates the type and species of gas generated through the heating of sediments, taking into account the influence of sediment type, heating temperature, and heating time. Following the exposition of these results, the argument is advanced that the climatic patterns observed in the rock record during multiple extinction events can be attributed to characteristic gas generation patterns resulting from combinations of slow and fast sediment heating at various temperatures. These distinct patterns, as identified through experimental data, serve as unique “fingerprints” contributing to our understanding of the nuanced causal factors involved in mass extinction events. The primary objective of this study is to illuminate the intricate details of these causal nuances, thereby enhancing our comprehension of the complex processes underlying mass extinction events.

**Figure 1:**
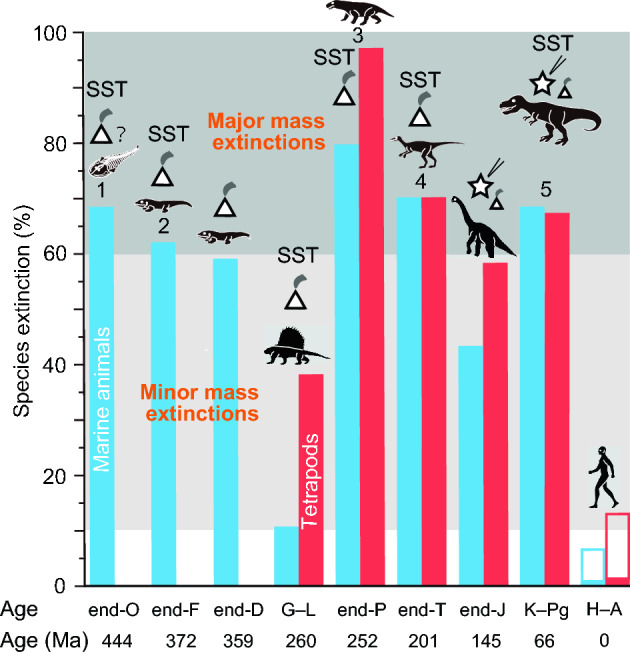
Percentages of species extinction in marine animals and tetrapods during major and minor mass extinctions. Blue columns indicate extinction values for marine species, while red columns represent extinction values for terrestrial species^4^. Open histograms in H–A depict species extinction percentages for 2060–2080 CE^53^. The abbreviations O, F, D, G–L, P, T, J, K–Pg, and H–A correspond to the following time periods: Ordovician, Frasnian, Devonian, Guadalupian–Lopingian transition, Permian, Triassic, Jurassic, Cretaceous–Paleogene boundary, Holocene–Anthropocene. These extinction percentage data are directly comparable due to the use of similar methods, including the conventional method and substage intervals^4^. The numbers 1 to 5 denote the five major mass extinctions. Each silhouette represents a representative vertebrate animal from the respective age. The end-Jurassic mass extinction event is associated with the Morokweng impact crater formation in South Africa^3^. SST: presence of SST data.

The release of sulfur dioxide (SO_2_) gas and soot into the stratosphere from volcanic eruptions and large meteorite impacts is known to induce global cooling, while the emissions of carbon dioxide (CO_2_) contribute to global warming. Several mass extinctions, such as those occurring at the end-Frasnian (end-F), end-Devonian (end-D), Guadalupian–Lopingian (G–L) transition, end-Permian (end-P), and end-Triassic (end-T) periods, have been linked to the specific volcanic activities of Large Igneous Provinces (LIPs)^1–3,5–12^. Multiple studies have substantiated the causal relationship between these extinction events and localized heating events^1–3,5–13^ (Fig. 1). Another significant extinction event took place at the Cretaceous–Paleogene (K–Pg) boundary, resulting from an asteroid impact at Chicxulub in the Yucatan Peninsula^13^, in conjunction with the volcanic activity of the Deccan Traps^14^. Heating events have also been confirmed in relation to this event^15,16^. Sea surface temperature (SST) data play a crucial role in comprehending the link between climate change and mass extinctions during these occurrences^4^.

Throughout the Phanerozoic, mass extinctions have displayed distinct climatic characteristics, with three instances linked to global cooling and two associated with global warming^4^. Kaiho et al.^11^ have proposed explanations for these differing outcomes in terms of cooling and warming. Through their rock heating experiments, they demonstrated that low-temperature heating (500–800 °C in 1–5-min heating^11^, 300–450 °C in 100-year heating^11^) results in high levels of SO_2_ emission from sulfide and low CO_2_ emissions, leading to long-term global cooling. On the other hand, medium-temperature (800–1000 °C in 1–5-min heating^11^, 450–550 °C in 100-year heating^11^) heating leads to high CO_2_ emissions from hydrocarbon and carbonates, causing global warming. This indicates that volcanic and impact heating of sedimentary rocks produces these gases depending on the temperature of heating.

In this study, I extend these findings by examining the temperature conditions necessary for SO_2_ gas formation from sulfate and the temperature-duration relationship for soot formation from organic matter. Through the compilation of data on heating temperature-duration, the coronene index (a measure of heating temperature)^9^, and surface temperature anomalies for each mass extinction event, my goal is to unveil the underlying processes that contribute to the distinct climates observed during mass extinctions.

## Results and discussion

### Effects of heating temperatures on gas production

**Figure 2:**
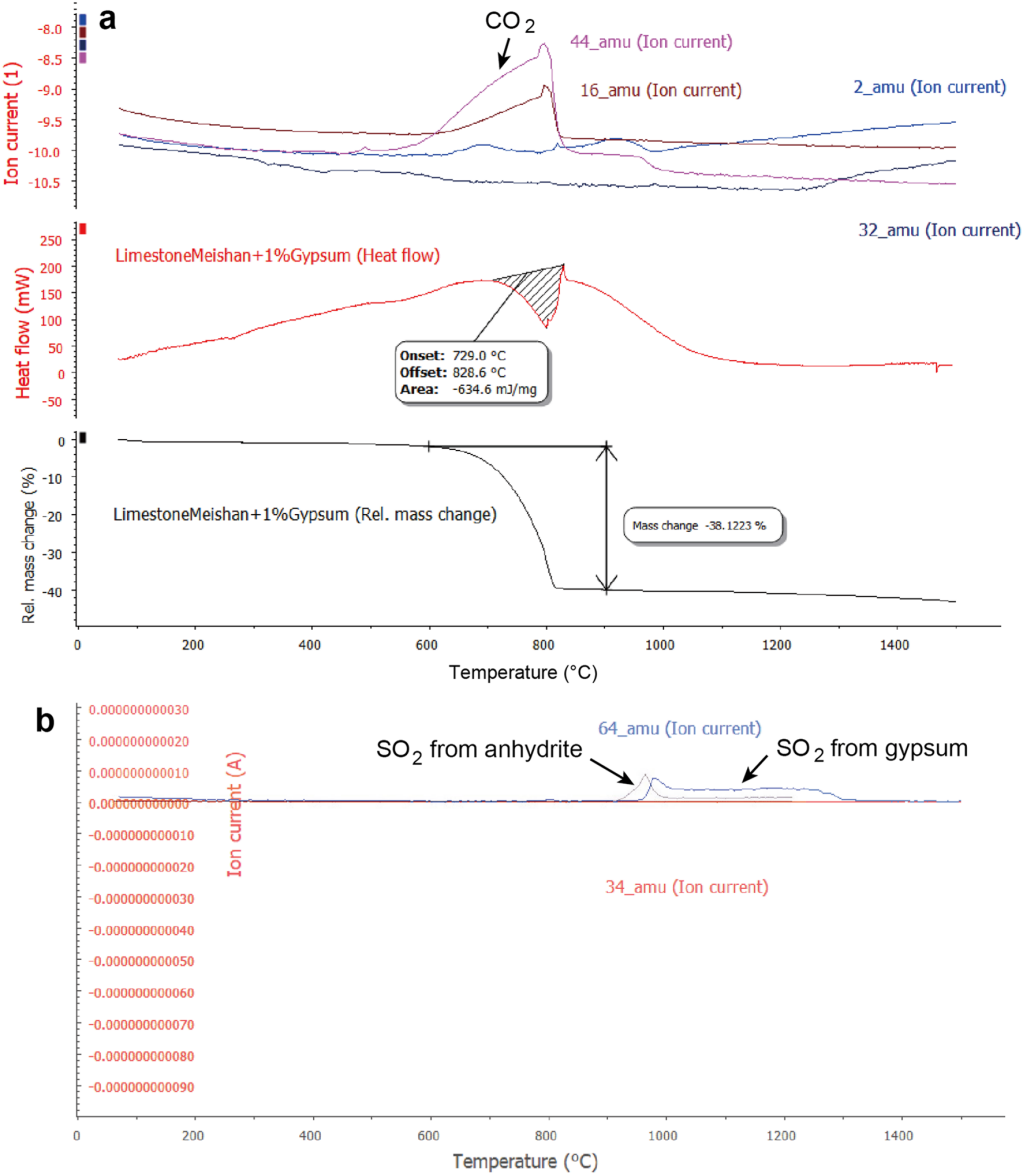
Temperature-dependent release of CO_2_ and SO_2_ gases from organic carbon-rich limestone containing 1 wt% sulfate (gypsum and anhydrite). (**a**) Temperature profiles illustrating the release of CO_2_ gas from organic carbon-rich limestone. (**b**) Temperature profiles illustrating the release of SO_2_ gas from samples containing 1 wt% gypsum (sulfate) and 1 wt% artificial anhydrite (sulfate). The curve labeled 44 amu represents the temperature for CO_2_ release, while the curve labeled 64 amu (**b**) corresponds to the temperature for SO_2_ release from gypsum and anhydrite. The molar ratio of SO_2_/g gypsum or anhydrite to CO_2_/g limestone is 4%, which corresponds to a weight ratio of 6%.

My analyses indicate that limestone samples with high organic carbon content exhibited significant CO_2_ emissions within the temperature range of 700–800 °C under slow heating at a rate of 10 °C/min (Fig. 2, represented by a red bar in Fig. 4). Additionally, SO_2_ gas was released from sulfate sources, namely gypsum and anhydrite, at temperatures ranging approximately from 1000 to 1300 °C (Fig. 2, depicted as a blue bar in Fig. 4). Whereas, SO_2_ gas was released from sulfide within the temperature range of 400–800 °C^17^ (a blue bar in Fig. 4). Combining these findings with previously published data from 1-, 10-, 100-, and 1000-min heating experiments (illustrated by red and blue dots in Fig. 4)^11^, these experimental data are categorized into three groups based on the sources of their formation: SO_2_ formed from sulfide under low-temperature heating, CO_2_ from hydrocarbons and carbonates under intermediate-temperature heating, and SO_2_ from sulfate under high-temperature heating (1000–1300 °C in 1–5-min heating, 550–650 °C in 100-year heating) (Fig. 4). The distribution of data in each group is represented by the Arrhenius equation, demonstrating the relationship between production temperature and heating time (refer to “Methods”). This alignment with the Arrhenius equation indicates that the experimental data conform to the chemical reaction role, as confirmed by the fit to the Arrhenius equation.

**Figure 3:**
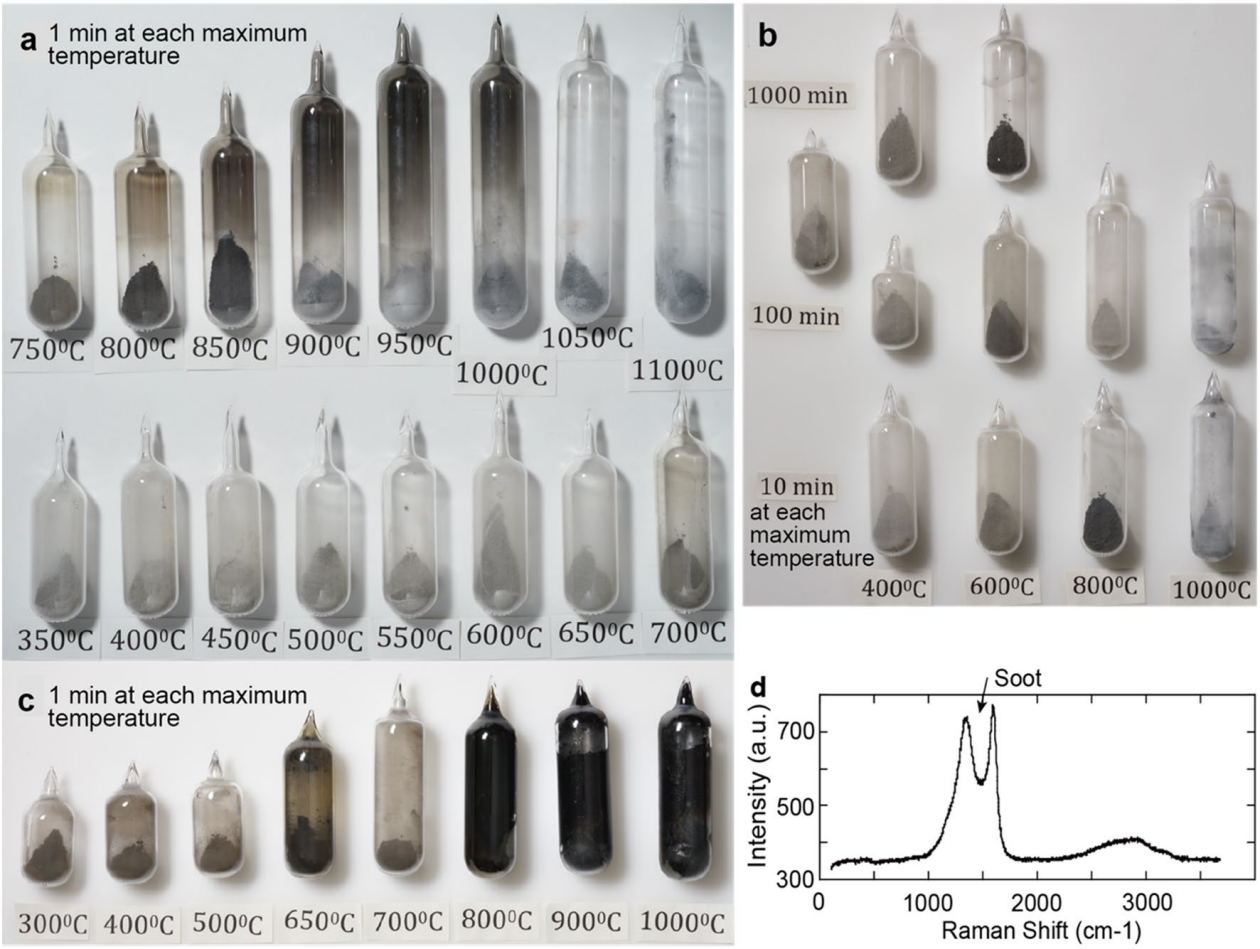
Temperature-dependent release of soot from sedimentary rocks, substantiated by compelling evidence. Temperature profiles illustrate the release of soot from organic carbon-rich limestone (**a**,**b**) and organic carbon-rich black shale (**c**). Refer to the “Methods” section for detailed information on samples and methodologies. During heating in panel (**a**) at 1050 °C and 1100 °C, the limestone powder underwent partial melting, indicating the conversion of CO_2_ to CO and O_2_, accompanied by the decomposition of soot. Importantly, this reaction is improbable in an open system. An examination of the Raman spectrum of a black material found on the inner wall of the ampoule confirmed its composition as soot (**d**). The outer diameter of the ampoules in panels (**a**,**b**) and panel (**c**) is 25 mm and 30 mm, respectively.

**Figure 4:**
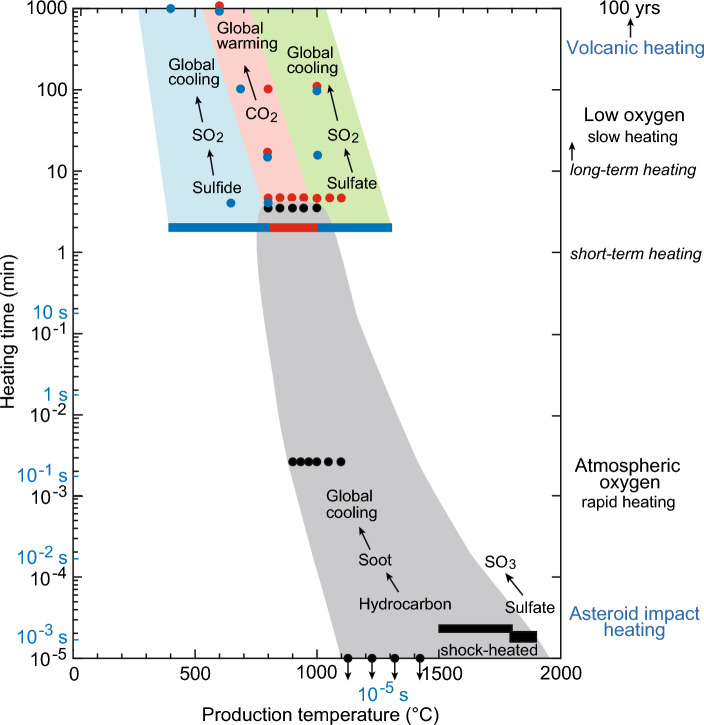
The temperature and heating durations required for the production of SO_2_, CO_2_, and soot were determined by integrating data from Figs. 2 and 3, along with findings from published papers. The figure illustrates the formation processes of various gases: SO_2_ from sulfide (based on Wang et al.^17^ using high-sulfur coal, with pyrolysis experiments conducted for 15 min at a rate of approximately 30 °C per minute, and Kaiho et al.^11^ using a heating rate of 8 °C per minute up to 1100 °C), CO_2_ from hydrocarbon and carbonate (derived from Kaiho et al.^11^ and the present study), SO_2_ from sulfate (from the present study), and soot from hydrocarbon (based on Krestinin et al.^19^ [isothermal pyrolysis of acetylene, benzene, and diacetylene], Yoshihara et al.^20^ under a shock-heated condition, He et al.^21^ under 2 and 4 atm shock temperature and pressure, model calculation of Chen et al.^22^, and the present study). The data points are represented by red and right-side blue bars, and black dots for 1-min heating, all obtained from the present study. The term “Low oxygen” corresponds to a pressure of 2 × 10^–4^ Pa. The colored areas on both sides of the frame, which encompass the data plots, represent the fitting of the experimental data to the Arrhenius equation. The Arrhenius equation elucidates the relationship between production temperature and heating time. The presence of experimental data points within these colored areas indicates how well they conform to the predicted behavior based on the Arrhenius equation. The production temperature on the horizontal axis indicates the heating temperature in each experiment.

These experimental processes and conditions marked by slow heating and low oxygen are suitable for simulating volcanism rather than asteroid impacts. Consequently, the findings suggest that substantial amounts of SO_2_ and CO_2_ gases are generated during the volcanic contact metamorphism of sedimentary rocks in Large Igneous Provinces (LIPs) through both low and high-temperature heating. Additionally, medium-temperature heating induces a significant release of CO_2_.

The heating temperatures employed in the brief laboratory experiments (ranging from a few seconds to a few days) were translated into equivalent temperatures aligned with the 100-year heating time scale, pertinent to sill contact metamorphism and derived from the Arrhenius equation (refer to the Supplementary Information in Kaiho et al.^11^). The maximum heating time of 100 years is based on a numerical model depicting the thermal evolution of a 100-m thick sill^18^. We postulate that during combustion, decomposition reactions involving calcium carbonate, pyrite (sulfide), and sulfate lead to the formation of carbon dioxide and oxidized sulfur species, respectively, depending on the heating temperature. These reactions were incorporated into the kinetics governing the time scale conversion. Consequently, this allows us to translate heating time from the experimental scale into a geologically relevant longer time scale^11^.

### Effects of heating temperatures on soot production

In the current analyses, soot formation was predominantly observed between 800 and 1000 °C, with each maximum temperature maintained for 1 min, in limestone and mudstone powder samples under a slow heating rate of 8 °C/min, at a low air pressure of 2 × 10^–4^ Pa (Fig. 3a,c). Raman analysis confirmed the presence of soot on the inside wall of the ampoules for the black materials (Fig. 3d). However, prolonged heating resulted in the disappearance of soot, with each maximum temperature maintained for 10–1000 min (Fig. 3b).

This study demonstrates that to initiate soot formation, a temperature range of 800–1000 °C is required under slow short-term heating and closed low air pressure conditions. Krestinin et al.^19^, who conducted experiments on the isothermal pyrolysis of acetylene and benzene with a pyrolysis time of 0.17 s, observed an increasing trend of soot formation from 900 to 1100 °C. Moreover, Yoshihara et al.^20^ showed that polyaromatic hydrocarbons (PAHs) and soot were formed at temperatures of 1500–1800 °C and 1800–1900 °C, respectively, within a time frame of 10^−3^ to 10^−2^ s under shock-heated conditions. He et al.^21^ demonstrated that abundant soot was formed at temperatures between 1500–1900 °C and at 2 and 4 atm shock temperature and pressure during 1.5 × 10^−3^ s. The model calculation of Chen et al.^22^ indicates soot formation under gas temperatures of 1100–1400 °C during 10^−5^ s.

To summarize, significant soot formation necessitates short-term heating, lasting less than a few minutes. The required temperature range is between 800–1000 °C under low air pressure conditions, resembling the process of volcanic eruptions. Similarly, temperatures between 900–1400 °C under atmospheric air pressure conditions during 10^−5^ to 10^−1^ s, and 1500–1900 °C under shock-heated conditions during 10^−3^ s resulting from rapid heating like an asteroid impact, also facilitate soot formation. These soot data distributions in the production temperature/heating time figure also fit the Arrhenius equation (Fig. 4). It is noteworthy that the asteroid impact scenario allows for soot formation across a wider temperature range. Contact metamorphism is the long-term heating, which cannot form soot as shown (Fig. 4). Volcanism can produce soot through the short-term heating process of volcanic activity. Consequently, the impact of volcanic soot on climate changes is relatively minor (Fig. 4).

### Heating temperatures and heating times for SO_2_, CO_2_, and soot production

Figure 4 illustrates the relationships between heating temperatures and heating times for the production of SO_2_, CO_2_, and soot. The data points in the figure conform to the distribution pattern predicted by the Arrhenius equation. This consistent pattern suggests that lower temperatures and longer heating times can produce SO_2_, CO_2_, and soot similarly to higher temperatures and shorter heating times, aligning with the principles of the Arrhenius equation. For instance, soot production at 1800–1900 °C during 10^−3^ s corresponds to that at 1000 °C during 1 min (Fig. 4). The variations in heating conditions are as follows: slow heating under low oxygen conditions corresponds to contact metamorphism by magma intrusion like a sill, whereas rapid heating under atmospheric oxygen conditions corresponds to impact heating.

**Figure 5:**
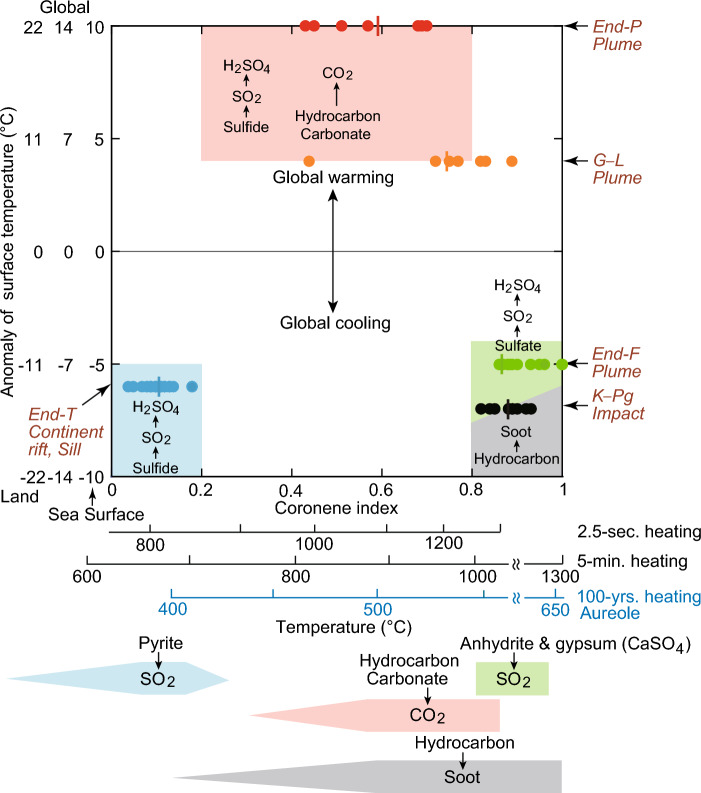
The three classifications of mass extinctions depicted in the upper part of this figure are based on surface temperature anomaly and the Coronene index (a sedimentary rock heating temperature index). The lower part of the figure illustrates the heating temperature based on experimental data and the major emission gases/particles influencing climate change. The colored vertical bars in the upper figure indicate the average values of the Coronene index for each mass extinction. Abbreviations and colors represent different events: End-F (end-Frasnian): green dots, G–L (Guadalupian–Lopingian transition): orange dots, End-P (end-Permian): red dots, End-T (end-Triassic): blue dots, and K–Pg (Cretaceous–Paleogene boundary): black dots. Specific emission gases in the lower figure, such as SO_2_, CO_2_, or soot, are determined by the heating temperature. The relationship between the three temperature scales connecting the upper and lower figures is based on the Arrhenius equation^11^. The upper temperature scale indicates the instantaneous heating temperature, such as that resulting from impact and experiments on the formation of PAHs, including coronene. While the temperature at the impact point exceeds 10,000 °C, the heating temperature decreases to a few hundred degrees moving from the center of the crater outward. The middle temperature scale represents the temperature during 5 min of heating based on Kaiho et al.^11^ and experimental data from this paper. The bottom temperature scale represents the temperature heated by sill according to Aarnes et al.^18^, lasting for 100 years.

**Figure 6:**
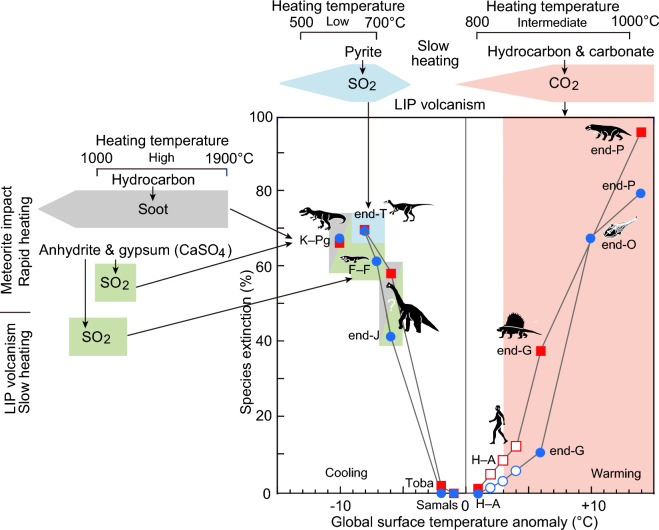
Relationship between heating temperature, global surface temperature anomaly, and extinction percentages. The color flames indicate the causes and outcomes of extinctions during specific periods: O: Ordovician, end-F: end-Frasnian, G: Guadalupian, P: Permian, T: Triassic, K–Pg: Cretaceous–Paleogene boundary, H–A: Holocene-Anthropocene. Toba: Toba volcanic eruption 74,000 years ago. Samals: Samals volcanic eruption in 1258 CE. Blue circles represent marine extinctions^4^, while red squares indicate terrestrial extinctions, represented by tetrapods^4^. In H–A, eight dots are from Kaiho^53^, with the six open dots representing projections for the near future (2060–2080 CE). Soot amount and surface temperature anomaly at the end-Jurassic were calculated^53^ and obtained using Figure 5 of Kaiho and Oshima^27^. Species extinction percentages for 2060–2080 CE are estimated based on 16 cases of global warming, environmental pollution, and forest devastation as causes^53^. The production temperature scale aligns with that in Fig. 5.

Figure 4 represents the primary outcome of this research. Leveraging the insights from Fig. 4 and incorporating the coronene index, elaborated upon in the next section, this paper has generated Figs. 5 and 6, which automatically present the conclusions. High-temperature heating resulting from the asteroid impact primarily released SO_3_ and minor amounts of SO_2_ (3% or more in the total amount of SO_2_ and SO_3_) from sulfates, leading to limited global cooling^23–26^. However, soot originating from hydrocarbons caused global cooling and played a role in the K–Pg mass extinction^15,27^.

### Grouping of mass extinctions based on coronene index values and global surface temperature anomalies

Coronene, a seven-ringed PAH, is produced during short-duration events under high temperatures^28^, such as asteroid impacts on target rocks^15^, hydrothermal volcanism^29^, and Large Igneous Province (LIP) volcanisms^9–12^. The anomalies in the coronene index and sea surface temperature (SST) can be attributed to volcanic or impact events (Table 1).

**Table 1:** Maximum surface temperature anomaly during mass extinction events.

Age	Age (Ma)	Coronene Index Average (SD P)	Observed global climate change	Temperature anomaly SST (°C)	Temperature anomaly Global (°C)	References
K–Pg boundary	66	0.88 (0.03)	Cooling	− 7	− 10	^15,51^
End-Triassic	201.56	0.11 (0.03)	Cooling	− 6	− 8	^11,49^
End-Permian	251.9	0.59 (0.11)	Warming	10	14	^9,52^
G–L transition	259	0.75 (0.13)	Warming	4	6	^12,50^
End-Frasnian	371.9	0.90 (0.04)	Cooling	− 5	− 7	^10,48^

Data from Table S1 and Kaiho et al.^9–12,15^.

SST and global surface temperature are shown in Kaiho^4^.

K–Pg: Cretaceous–Paleogene.

G–L: Guadalupian–Lopingian.

Sedimentary rocks with low coronene index values (0–0.2) typically indicate low-temperature heating, corresponding to the end-Triassic mass extinction^11^. These values align with background values for mass extinctions and averaged wildfire temperatures^9–12,15^ (Supplementary Table 1, Table 1). During the onset of the end-Triassic mass extinction, sedimentary rock samples from Austria (including the Global Boundary Stratotype Section and Point [GSSP]) and the UK exhibited low coronene index values (average 0.11 [SD 0.03]) (Table 1)^11^.

Intermediate coronene index values (0.2–0.8), indicating medium-temperature heating (700–1000 °C for 1 min), were observed in volcanic-generated mass extinctions at the Guadalupian–Lopingian transition in South China (including GSSP; average 0.75 [SD 0.13])^12^, and at the end of the Permian in South China (including GSSP and a section ~ 1000 km away) and Italy (average 0.59 [SD 0.11])^9^.

High coronene index values (0.8–1.0) were found at the end of the Frasnian in France and Belgium (including GSSP; average 0.90 [SD 0.04])^10^, as well as the impact-generated mass extinction at the K–Pg boundary in Haiti and Spain (average 0.88 [SD 0.03])^15^ (Fig. 5).

For the four events except for G–L, coronene index values are distinctly concentrated to low at End-T, medium at end-P, and high at end-F and K–Pg, resulting in no overlapping between the low, medium, and high values. While G–L has a wide range from 0.4 to 0.9, it does not plot in the low-temperature range.

The global surface temperature anomalies during each mass extinction were summarized in Kaiho^4^. Based on the surface temperature anomalies and the coronene index, these five mass extinctions can be grouped into three categories: (i) global cooling and low coronene index observed at the end of the Triassic, (ii) global warming and intermediate coronene index observed at the G–L transition and the end of the Permian, and (iii) global cooling and high coronene index at the end of the Frasnian and K–Pg boundary (Fig. 5, Table 1).

### Determining factors of heating temperature

The presence of extremely high temperatures, surpassing 10,000 °C, at the center of impact craters explains the observed high coronene index at the K–Pg boundary^15^. In contrast, the heightened heating temperature at the end-Frasnian can be attributed to plume magma volcanism within Large Igneous Provinces (LIPs), deep sills, and older-age magma (Table 2). These volcanic events, characterized by explosive carbonatite-alkaline magmatism and associated kimberlite-type eruptions, exhibit temperatures around 1400 °C in the Late Devonian, as determined from the age/temperature curve of Herzberg et al.^30^. Covering extensive areas with estimated diameters ranging in the thousands of kilometers, such intense volcanic activity has the potential to generate a high fraction of coronene, a seven-ringed PAH, through the significant heating of large volumes of sedimentary hydrocarbons present in older strata^10^.

**Table 2:** Coincidence of mass extinctions and heating events.

Mass extinction	Age (Ma)	Location of heating	Age (Ma) of heating	Cause	References
K–Pg	66	Chicxulub	Impact: 66.0	Meteorite impact	^13–15,54,54–61^
End-Jurassic	201.56	CAMP	Sill: 201.6–201.0	Continent rift-related volcanism	^11,62–67^
End-Permian	251.9	Siberian Trap	Pyroclastic, sill: 251.9	Plume volcanism	^1,9,34^
G–L	259	Emeishan	Main phase: 260.9–259.1	Plume volcanism	^8,68–71^
End-F	371.9	Yakutsk-Viluy LIP	Eruption: 374 ± 3.5	Plume volcanism*	^7,37,39^
		PDD LIP	Rift structure: 377–370	Plume volcanism*	^7,38^

CAMP: Central Atlantic Magmatic Province.

PDD: Pripyat–Dnieper–Donets.

*Carbonatite/kimberlite.

However, low-temperature heating at the end-Triassic mass extinction is attributed to a shallower mantle source, shallower sill depths, and younger mantles, exhibiting temperatures around 1370 °C in the end-Triassic (Table 3). Medium-temperature heating at the end-Permian mass extinction is due to a deep mantle source, medium sill depths, and around 1370 °C mantle temperature.

**Table 3:** Lithologies of large igneous provinces for five mass extinctions and asteroid impact target rocks.

Location	Age	Main lithology	Minor lithology	Thickness (km)	Sill	References
Chicxulub	K–Pg	Carbonate, evaporite	Shale, oil	3		^15,27,40^
CAMPS	End-Jurassic	Carbonate, evaporite	Clastic rocks	3–4	Yes	^36^
Siberian Trap	End-Permian	Marl, carbonate, evaporite	Shale, sandstone, coal, oil	3–5	Yes	^1,34^
Emeishan	G–L	Carbonate	Clastic rocks	?	Yes	^8^
Yakutsk-Viluy LIP	End-F	Carbonate, evaporite	Shale	7–8	Yes	^37,39^
PDD LIP	End-F	Carbonate, evaporite, marl	Shale	6–6.5	Yes	^7,38^

Thickness: Thickness of sedimentary rocks heated by sills-dykes-magmas or impact.

The presence of high coronene index values cannot be solely attributed to wildfires. Previous studies have indicated that the abundance of coronene and benzo[ghi]perylene, which are derived from petroleum sediments, can be associated with hydrothermal volcanism off the coast of California^29^. Thus, volcanism can contribute to the formation of six to seven-ring PAHs, including coronene. Although forest fires frequently occur during the deposition of sediments in seas, it is important to note that during non-event periods, the coronene index values are typically less than 0.2^9–12,15^. High-temperature wildfires, such as the recent ones witnessed in Australia with temperatures reaching 1200 °C, can contribute to the formation of coronene. Sedimentary rocks found in seas contain PAHs derived from numerous wildfires occurring in various regions dominated by forests and grasslands. The temperature of a typical grassland wildfire is around 300 °C, leading to a low coronene index when PAHs from different types of wildfires mix.

The ratio of the sum of benzo[e]pyrene, benzo[ghi]perylene, and coronene to phenanthrene (5- to 7-ring PAH index) is used as an indicator of heating events^9–11,31^. The 5- to 7-ring PAH index is significantly elevated in heating events and mass extinctions compared to their background values (non-extinction periods)^9–11^. While the coronene index values during the end-Triassic mass extinction are similar to the background values, the 5- to 7-ring PAH index is higher. This indicates that the average heating temperature during the mass extinctions, including the end-Triassic low-temperature volcanic event, was higher than the average temperature of wildfires.

These PAH events include large-scale volcanism^9–11^, meteorite impact events^15,32^, and hydrothermal systems^29,33^. The preference for these events is due to their higher energy requirements for PAH formation^28^. The concentration of coronene or benzo[ghi]perylene in six mass extinctions (end-Frasnian, end-Devonian, G–L transition, end-Permian, end-Triassic, and K–Pg boundary) supports the simultaneous occurrence of large-scale volcanic activity or asteroid impact and mass extinction (Table 2)^9–12^.

### The impact of heating temperature on global climate

The correlation between coronene index values and gas production temperature has been analyzed using the heating experimental data on PAHs from Norinaga et al.^28^ (Fig. 5). This correlation suggests that the grouping of mass extinctions based on coronene index values and global surface temperature anomalies can be associated with heating temperatures/time for gas and soot production.

Global climate changes appear to be influenced by the temperature, volume, and composition of sedimentary rocks in Large Igneous Provinces (LIPs), providing a mechanism to explain the diverse climate changes resulting from LIP volcanism^11,34^. Soot formation is observed during short-term heating, characteristic of impacts, while long-term heating in volcanic settings typically leads to the production of SO_2_ and CO_2_ unless there is a sudden release of heated hydrocarbons through eruptions. These SO_2_ and soot must reach the stratosphere to form sulfuric acid and soot aerosols inducing global cooling, a process facilitated by the high energy of volcanic eruptions and asteroid/comet impacts. When soot formed from impact target rocks was introduced into the stratospheric SO_2_ from the Deccan volcanism, the soot blocked sunlight, preventing the formation of sulfuric acid from SO_2_ due to reduced sunlight.

Large Igneous Provinces (LIPs), with diameters ranging from 1000 to 2000 km, exhibit similarities in rock types and proportions, primarily composed of carbonates, evaporites, and clastic rocks^1,7,8,34–39^ (Table 3). The composition of the impact target rocks at Chicxulub also includes carbonates, evaporites, and clastic rocks^40^. These suggest that there is likely minimal bias in how emitted gases, soot, and coronene are recorded between different events. The gases and soot released from LIPs and impact target rocks exist in the amount necessary to cause global climate changes^4^, as shown in Svensen et al.^34^ for the Siberian LIP, Kaiho et al.^11^ for CAMP, and Kaiho and Oshima^27^ for the Chicxulub impact. Many sills are present, as shown in Table 3 (thickness, see note) for all Lips.

The heating temperature significantly influences the amount of released SO_2_ and CO_2_, resulting in either cooling or warming effects. The sulfate content in evaporites may vary; nevertheless, sulfate does not dissolve at low to medium temperatures, impeding the release of SO_2_ gas from sulfate. In cases such as the K–Pg and end-F, the high heating temperature generated SO_2_ gas from sulfate rocks abundant in sulfate.

Sulfides are present in substantial amounts in all sedimentary rocks, approximately 100 times that of sulfate, resulting in the emission of SO_2_ gas at low, medium and high temperatures across various LIPs. The common occurrence of hydrocarbons and carbonates in target rocks for impacts and LIPs leads to significant CO_2_ generation at both moderate and high temperatures^11^. Consequently, low-temperature heating scenarios release SO_2_ from sulfides, dominating stratospheric sulfate aerosols and causing long-term global cooling. Conversely, in moderate-temperature heating scenarios, the predominant release of CO_2_ from hydrocarbons induces global warming.

In carbonate rocks with small amounts of sulfate, high-temperature heating contributes to global cooling. Locations abundant in evaporites, specifically sulfate rocks, experience more pronounced cooling, surpassing the greenhouse effect of CO_2_. For instance, the end-F LIPs, characterized by abundant sulfate rocks, exhibit significant cooling due to the sunlight-blocking effect of sulfate aerosols.

The asteroid impact at the K–Pg boundary, causing short-term heating, leads to the generation of soot from hydrocarbons in sedimentary rocks. This induces global cooling irrespective of the quantity of sulfate rocks.

The interaction between magmas and sedimentary rocks results in the significant release of SO_2_ and CO_2_ gases through sill contact metamorphism, which in turn leads to diatreme pipe eruptions^1,34^. Evidence of contact metamorphism has been documented in association with mass extinctions in the end-F, D-C, end-Permian, and end-Triassic, with numerous sills reported across a wide range^1,7,34–36,41,42^ (see Table 3). The quantity of sills and sedimentary rocks in contact necessary to trigger global changes is present within each LIP^1,7,34–36,41,42^. Utilizing Arrhenius's equation, the experimental temperature over minutes to one day is converted to a temperature scale spanning 100 years for sills. This temperature correlates with the temperature of the aureole surrounding the sill.

Examining specific mass extinction events, such as the end-Triassic, end-Permian, and Late Devonian, reveals distinct patterns of SO_2_ and CO_2_ emissions corresponding to low, intermediate, and high-temperature heating, influencing global cooling or warming. The Chicxulub impact at the K–Pg boundary, characterized by short-term heating, released substantial quantities of SO_3_, soot, and CO_2_ gases from sedimentary rocks, contributing to global cooling^15,27^.

Global cooling resulting from a single volcanic eruption or impact event occurs over 2–3 years, mostly recovering within approximately 10 years^15^. Subsequently, a period of warming follows^43^. The prominence of either cooling or warming depends on the heating temperature. Specifically, large-scale volcanic regions with a diameter of approximately 2000 km, containing diverse sedimentary rocks, establish heating temperatures as a key factor in climate change dynamics.

### The influence of heating temperature on mass extinctions

Our analysis underscores the crucial role of sedimentary rock temperature in shaping climate changes during mass extinctions. We have identified distinct temperature-dependent processes that drive global cooling and warming. Low-temperature (400–750 °C) heating of sulfide leads to the release of SO_2_, triggering global cooling through the formation of sulfuric acid aerosols. This phenomenon was observed during sill development in the continental rift volcanism of Central Atlantic Magmatic Province. Central Atlantic Magmatic Province volcanism, which contributed to the end-Triassic mass extinction (Fig. 6). Intermediate temperature (700–1100 °C) heating of hydrocarbons and carbonates releases significant amounts of CO_2_, resulting in global warming. Such occurrences were observed during felsic extrusive volcanic eruptions in the Emeishan and Siberian large igneous provinces, leading to the G–L mass extinction and end-Permian mass extinction, respectively (Fig. 6). High-temperature (1000–1300 °C) heating produces both SO_2_ from sulfate and sulfide, leading to global cooling. Notably, this was observed during ultrabasic kimberlite-like eruptions associated with the onset of the end-Frasnian mass extinction. Furthermore, at medium–high temperatures and short heating durations, the formation of soot from hydrocarbons plays a significant role in global cooling.

## Conclusions

This study focused on the mechanisms of climate change during mass extinctions in the Phanerozoic Eon. These variations include cases where global cooling is predominant and cases where global warming is predominant, but the reasons for these are not always clear for each mass extinction event. I showed that the temperature to which sedimentary rocks are heated determines these climate changes. Low-temperature heating of sulfides causes global cooling through the release of sulfur dioxide (SO_2_), while intermediate-temperature heating of hydrocarbons and carbonates results in the release of large amounts of carbon dioxide (CO_2_), leading to global warming. Furthermore, high-temperature heating generates SO_2_ from sulfates, contributing to global cooling. In the case of large-scale volcanic activity, magma intrudes into sedimentary rocks, generating the aforementioned volatile gases depending on the temperature of contact heating. These gases are then released into the atmosphere through gas explosions, causing the aforementioned climate changes. Additionally, short-term medium to high-temperature shock heating resulting from asteroid impacts generates soot from hydrocarbons, also contributing to global cooling.

To further clarify the mechanisms underlying climate control through the heating temperature of sedimentary rocks during significant mass extinctions, future investigations should prioritize the acquisition of data pertaining to the following:

1. Sea surface temperature (SST) during the end-Devonian mass extinction and end-Jurassic mass extinction (Fig. 6).
2. Coronene index and the quantities of five- to seven-ring PAHs during the end-Ordovician mass extinction and end-Jurassic mass extinction (Fig. 5).

By obtaining these additional data sets, we can develop a more comprehensive understanding of the role of heating temperature in driving climate changes during mass extinctions.

This paper has elucidated the causal mechanisms of climate change associated with five well-documented mass extinctions. It has achieved this by clarifying the relationship between heating temperature, the release amount of SO_2_, CO_2_, and soot, which control global climate, and the heating temperature proxy, coronene index, recorded in sedimentary rocks. Additionally, the study reveals the evident connection between climate change itself and its causal locations.

## Methods

### Determination of CO_2_ and SO_2_ gas release temperature

This study aims to determine the temperatures at which CO_2_ and SO_2_ gases are released from organic carbon-rich limestone, specifically those containing 1 wt% gypsum (sulfate) and 1 wt% anhydrite (sulfate), respectively. The experiments were conducted using the TG/STA (thermo balance) coupled with a Quadrupole Mass Spectrometer (QMS) system (Fig. 2). The rock sample utilized in the experiments is a Changhsingian limestone sourced from Meishan, China, containing a total organic carbon (TOC) content of 0.5%, supplemented by Miocene gypsum obtained from San Jose, California and artificial anhydrite. The heating temperature during the experiments was gradually increased by 10 °C within a duration of 1 min, up to 1500 °C. This approach allowed for controlled heating of the sedimentary rocks and the measurement and characterization of gas release. The relevant data are presented in Fig. 2. In this study, we also utilize previous gas release measurements, specifically the type and quantity of gas released from heated mudstone and limestone^11^, as well as high-sulfur coal^17^, to determine the temperature at which CO_2_ and SO_2_ gases are released.

### Investigation of soot formation temperature

In addition to the aforementioned experiments, heating experiments were performed on sedimentary rocks to examine soot formation. Raman analyses were employed to identify the presence of soot. Sixteen powder samples were prepared from the previously mentioned limestone (Fig. 3a, b), while eight powder samples were prepared from a Changhsingian black shale from Shangsi, China, with a higher total organic carbon (TOC) content of 2.0% (Fig. 3c). For each rock sample, varying amounts of powder (1, 2, or 5 g)^11^ were placed into quartz ampoules, which were then evacuated and sealed at a pressure of 2 × 10^−4^ Pa. Each ampoule was individually heated in an electric furnace, with temperatures ranging from 350 to 1100 °C in increments of either 50 °C or 100 °C^11^. Each maximum temperature is kept for 1 min to 1000 min (Fig. 3a–c). The maximum temperature within ± 10 °C is kept for approximately 5 min in the 1-min case. Five minutes is used as heating time for soot in the 1-min case (Fig. 3).

### Arrhenius equation

To determine the frames of experimental data in Fig. 4, we utilized an equation derived from the Arrhenius equation: T_1_ = E_a_T_2_/(E_a_ − T_2_ * ln[t_2_/t_1_]); T_1_: temperature in actual heating time; Ea: activation energy (74 kcal/mol for *n*-C_16_ alkane^44^; 74 kcal/mol for calcium carbonate^45^ [310 kJ/mol = 74 kcal/mol], and 67 kcal/mol for pyrite^46^; T_2_: temperature in the experiment; t_1_: heating time (s) in the experiment; t_2_: actual heating time^11^. There are no significant differences in amount of hydrocarbon and CO_2_ gases generated from under different pressure between 0.1 MPa (1 atm) and 30 MPa (1 km depth underground, which is maximum depth treated in this study) based on experiment data of oil and coal combustion^11,44,47^. Pressure difference on average causes ca. 10 °C higher, which can be neglected^11,44^.

### Compilation of SST and coronene index

The analysis in this study focuses exclusively on sea surface temperature (SST) data from low latitudes, obtained from sources^48–52^. It is important to note that surface temperature anomalies at low latitudes consistently exhibit intermediate values, indicating proximity to average values^4^. The error margin for SST anomalies in geological ages is approximately 1 °C, which includes approximately ± 0.5 °C depending on the sample location to obtain the average value and approximately ± 0.5 °C depending on the detection of the largest anomaly for abrupt short-term events from sedimentary rocks, typically deposited at a rate of 1–100 mm kyr^−1^, except for impact ejecta sediment^4^. SST anomalies from various geological ages were converted to global surface temperature anomalies and land-surface temperature anomalies using Figure 1d of Kaiho^4^, which was generated from global cooling and warming (recovery) data obtained through climate model calculations^27^. These compiled data are presented in Table 1.

The coronene index, proposed by Kaiho et al.^9^, is defined as the ratio of coronene (seven-ringed polycyclic aromatic hydrocarbons [PAH]) to the sum of benzo[e]pyrene (five-ringed PAH), benzo[ghi]perylene (six-ringed PAH), and coronene itself. It serves as an indicator of heating temperature. Previous studies^9–12,31^ have utilized this index to investigate mass extinction events. The coronene index data are compiled in Table 1.

Based on these findings, this paper proposes a theory that seeks to uncover the mechanisms responsible for the observed global cooling and warming during mass extinctions by synthesizing the summarized data.

### Supplementary Information

Supplementary Information.

## Data Availability

All data is available in the main text or the supplementary materials.

## References

[1] Svensen H (2009). Siberian gas venting and the end-Permian environmental crisis. Earth Planet. Sci. Lett..

[2] Bond DPG, Grasby SE (2017). On the causes of mass extinctions. Palaeogeogr. Plaeoclimatol. Palaeoecol..

[3] Rampino MR, Caldeira K, Prokoph A (2019). What causes mass extinctions? Large asteroid/comet impacts, flood-basalt volcanism, and ocean anoxia—Correlations and cycles. Geol. Soc. Am. Spec. Pap..

[4] Kaiho K (2022). Relationship between extinction magnitude and climate change during major marine and terrestrial animal crises. Biogeosciences.

[5] Burgess SD, Muirhead JD, Bowring SA (2017). Initial pulse of Siberian Traps sills as the trigger of the end-Permian mass extinction. Nat. Commun..

[6] Heimdal TH (2019). Evidence for magma-evaporite interactions during the emplacement of the Central Atlantic Province (CAMP) in Brazil. Earth Planet. Sci. Lett..

[7] Racki G (2020). A volcanic scenario for the Frasnian-Famennian major biotic crisis and other Late Devonian global changes: More answers than questions?. Glob. Planet. Change.

[8] Zhong Y (2020). Geochemical, biostratigraphic, and high-resolution geochronological constraints on the waning stage of Emeishan Large Igneous Province. Geol. Soc. Am. Bull..

[9] Kaiho K, Aftabuzzaman M, Jones DS, Tian L (2021). Pulsed volcanic combustion events coincident with the end-Permian terrestrial disturbance and the following global crisis. Geology.

[10] Kaiho K (2021). Coronene, mercury, and biomarker data support a link between extinction magnitude and volcanic intensity in the Late Devonian. Glob. Planet. Change.

[11] Kaiho K (2022). Volcanic temperature changes modulated volatile release and climate fluctuations at the end-Triassic mass extinction. Earth Planet. Sci. Lett..

[12] Kaiho K, Grasby SE, Chen Z-Q (2023). High-temperature combustion event spanning the Guadalupian−Lopingian boundary terminated by soil erosion. Palaeogeogr. Plaeoclimatol. Palaeoecol..

[13] Schulte P (2010). The Chicxulub asteroid impact and mass extinction at the Cretaceous-Paleogene boundary. Science.

[14] Sprain CJ (2019). The eruptive tempo of Deccan volcanism in relation to the Cretaceous-Paleogene boundary. Science.

[15] Kaiho K (2016). Global climate change driven by soot at the K–Pg boundary as the cause of the mass extinction. Sci. Rep..

[16] Lyons SL (2020). Organic matter from the Chicxulub crater exacerbated the K–Pg impact winter. Proc. Natl. Acad. Sci. USA.

[17] Wang M (2019). Effect of steam on the transformation of sulfur during demineralized coal pyrolysis. J. Anal. Appl. Pyrolysis.

[18] Aarnes I, Svensen H, Connolly J, Podladchikov YY (2010). How contact metamorphism can trigger global climate changes: Modeling gas generation around igneous sills in sedimentary basins. Geochim. Cosmochim. Acta.

[19] Krestinin AV, Kislov MB, Raevskii AV, Kolesova OI, Stesik LN (2000). On the mechanism of soot particle formation. Kinet. Catal..

[20] Yoshihara Y, Ikegami M, Natake S (1994). Kinetics of soot cluster formation at high temperatures. JSME Int. J. Ser. B.

[21] He J (2017). Experimental study of the soot formation of RP-3 behind reflected shock waves. Fuel.

[22] Chen Y, Zhang J (2013). Analysis of the influences of gas temperature fluctuation on the soot formation and oxidation. Fuel.

[23] Gerasimov, M. V., Dikov, Y. P., Yakovlev, O. I. & Wlotzka, F. High-temperature vaporization of gypsum and anhydrite: Experimental results (Abstr.). *Lunar Planet. Sci. Conf.* XXV, 413–414 (Lunar Planet. Inst., 1994).

[24] Badjukov, D. D., Dikov, Y. P., Petrova, T. L. & Pershin, S. V. Shock behavior of calcite, anhydrite. and gypsum (Abstr.). *Lunar Planet. Sci. Conf. XXVI* 63–64 (Lunar Planet. Inst., 1995).

[25] Ivanov BA (1996). Degassing of sedimentary rocks due to Chicxulub impact: Hydrocode and physical simulations. Geol. Sot. Am. Spec. Pap..

[26] Ohno S (2014). Production of sulphate-rich vapour during the Chicxulub impact and implications for ocean acidification. Nat. Geosci..

[27] Kaiho K, Oshima N (2017). Site of asteroid impact changed the history of life on Earth: The low probability of mass extinction. Sci. Rep..

[28] Norinaga K, Deutschmann O, Saegusa N, Hayashi JI (2009). Analysis of pyrolysis products from light hydrocarbons and kinetic modeling for growth of polycyclic aromatic hydrocarbons with detailed chemistry. J. Anal. Appl. Pyrolysis.

[29] Simoneit BR, Fetzer JC (1996). High molecular weight polycyclic aromatic hydrocarbons in hydrothermal petroleums from the Gulf of California and Northeast Pacific Ocean. Org. Geochem..

[30] Herzberg C, Condie K, Korenaga J (2010). Thermal history of the Earth and its petrological expression. Earth Planet. Sci. Lett..

[31] Saito R, Kaiho K, Tian L, Takahashi S (2023). Frequent high-temperature volcanic combustion events delayed biotic recovery after the end-Permian mass extinction. Earth Planet. Sci. Lett..

[32] Hallmann C, Grey K, Webster LJ, McKirdy DM, Grice K (2010). Molecular signature of the Neoproterozoic Acraman impact event. Org. Geochem..

[33] Simoneit BRT, Lein AY, Peresypkin VI, Osipov GA (2004). Composition and origin of hydrothermal petroleum and associated lipids in the sulfide deposits of the Rainbow field (Mid-Atlantic Ridge at 36°N). Geochim. Cosmochim. Acta.

[34] Svensen H (2018). Sills and gas generation in the Siberian traps. Philos. Trans. R. Soc. A.

[35] Davies JHFL (2017). End-Triassic mass extinction started by intrusive CAMP activity. Nat. Commun..

[36] Heimdal TH (2018). Large-scale sill emplacement in Brazil as a trigger for the end-Triassic crisis. Sci. Rep..

[37] Shpount BR, Oleinikov A, Halls HC, Fahrig WF (1987). A comparison of mafic dike swarm from the Siberian and Russian platforms. Mafic Dyke Swarms, Special Paper, 34.

[38] Makhnach A, Mikhajlov N, Shimanovich V, Kolosov I (1994). Carbon and oxygen isotopic composition of carbonates from saliferous deposits of the Pripyat Trough. Belarus. Sediment. Geol..

[39] Kravchnsky (2002). Palaeomagnetism of East Siberian traps and kimberlites: Two new poles and palaeogeographic reconstructions at about 360 and 250 Ma. Geophys. J. Int..

[40] Robertson DS, Lewis WM, Sheehan PM, Toon OB (2013). K–Pg extinction: Reevaluation of the heat-fire hypothesis. J. Geophys. Res. Biogeoscience.

[41] Ricci J (2013). New ^40^Ar/^39^Ar and K-Ar ages of the Viluy traps (Eastern Siberia): Further evidence for a relationship with the Frasnian-Famennian mass extinction. Palaeogeogr. Palaeoclimatol. Palaeoecol..

[42] Courtillot V, Kravchinsky VA, Quidelleur X, Renne PR, Gladkochub DP (2010). Preliminary dating of the Viluy traps (Eastern Siberia): Eruption at the time of Late Devonian extinction events?. Earth Planet. Sci. Lett..

[43] Black BA (2018). Systemic swings in end-Permian climate from Siberian Traps carbon and sulfur outgassing. Nat. Geosci..

[44] Jackson KJ, Burnham AK, Braun RL (1995). Temperature and pressure dependence of n-hexadecane cracking. Org. Geochem..

[45] Fedunik-Hofman L, Bayon A, Donne SW (2019). Comparative kinetic analysis of CaCO_3_/CaO reaction system for energy storage and carbon capture. Appl. Sci..

[46] Concer PH (2017). Kinetics of the oxidation reactions and decomposition of pyrite. Ceramica.

[47] Mi J, Zhang S, He K (2014). Experimental investigations about the effect of pressure on gas generation from coal. Org. Geochem..

[48] Balter V, Renaud S, Girard C, Joachimski MM (2008). Record of climate-driven morphological changes in 376Ma Devonian fossils. Geology.

[49] Korte C, Hesselbo SP, Jenkyns HC, Rockaby RE, Spoetl C (2009). Palaeoenvironmental significance of carbon- and oxygen-isotope stratigraphy of marine Triassic-Jurassic boundary sections in SW Britain. J. Geol. Soc..

[50] Chen B, Joachimski MM, Sun YD, Shem SZ, Lai XL (2011). Carbon and conodont apatite oxygen isotope records of Guadalupian-Lopingian boundary sections: Climatic or sea-level signal?. Palaeogeogr. Palaeoclimatol. Palaeoecol..

[51] Vellekoop J (2014). Rapid shortterm cooling following the Chicxulub impact at the Cretaceous-Paleogene boundary. Proc. Natl. Acad. Sci. USA.

[52] Chen J (2016). High-resolution SIMS oxygen isotope analysis on conodont apatite from South China and implications for the end-Permian mass extinction. Palaeogeogr. Palaeoclimatol. Palaeoecol..

[53] Kaiho K (2023). An animal crisis caused by pollution, deforestation, and warming in the late 21st century and exacerbation by nuclear war. Heliyon.

[54] Chiarenza AA (2020). Asteroid impact, not volcanism, caused the end-Cretaceous dinosaur extinction. Proc. Natl. Acad. Sci. USA.

[55] Alvarez LW, Alvarez W, Asaro F, Michel HV (1980). Extraterrestrial cause for the Cretaceous-Tertiary extinction. Science.

[56] Archibald JD (2010). Cretaceous extinctions: Multiple causes. Science.

[57] Sial AN (2016). Mercury enrichment and Hg isotopes in Cretaceouse-Paleogene boundary successions: Links to volcanism and palaeoenvironmental impacts. Cretaceous Res..

[58] Schoene B (2019). U-Pb constraints on pulsed eruption of the Deccan Traps across the end-Cretaceous mass extinction. Science.

[59] Hull PM (2020). On impact and volcanism across the Cretaceous-Paleogene boundary. Science.

[60] Keller G (2020). Mercury linked to Deccan Traps volcanism, climate change and the end-Cretaceous mass extinction. Glob. Planet. Change.

[61] Font E, Chen J, Regelous M, Regelous A, Adatte T (2022). Volcanic origin of the mercury anomalies at the Cretaceous-Paleogene transition of Bidart, France. Geology.

[62] Schoene B, Guex J, Bartolini A, Schaltegger U, Blackburn TJ (2010). Correlating the end-Triassic mass extinction and flood basalt volcanism at the 100 ka level. Geology.

[63] Blackburn TJ (2013). Zircon U-Pb geochronology links the end-Triassic extinction with the Central Atlantic Magmatic Province. Science.

[64] Marzoli A (2004). Synchrony of the Central Atlantic magmatic province and the Triassic-Jurassic boundary climatic and biotic crisis. Geology.

[65] Marzoli A (2019). Massive methane fluxing from magma–sediment interaction in the end-Triassic Central Atlantic Magmatic Province. Nat. Commun..

[66] Lindström S (2021). Tracing volcanic emissions from the Central Atlantic Magmatic Province in the sedimentary record. Earth Sci. Rev..

[67] Capriolo M (2021). Massive methane fluxing from magma-sediment interaction in the End-Triassic Central Atlantic Magmatic Province. Nat. Commun..

[68] Zhong YT, He B, Mundil R, Xu YG (2014). CA– TIMS zircon U-Pb dating of felsic ignimbrite from the Binchuan section: Implications for the termination age of Emeishan large igneous province. Lithos.

[69] Yang JH (2018). Early Wuchiapingian cooling linked to Emeishan basaltic weathering?. Earth Planet. Sci. Lett..

[70] Huang H (2022). Eruptive tempo of Emeishan large igneous province, southwestern China and northern Vietnam: Relations to biotic crises and paleoclimate changes around the Guadalupian-Lopingian boundary. Geology.

[71] Chen J, Xu YG (2019). Establishing the link between Permian volcanism and biodiversity changes: Insights from geochemical proxies. Gondwana Res..

