# Supplementary material for: Role of volcanism and impact heating in mass extinction climate shifts
Source: Sci Rep. 2024 Apr 30;14:9946. doi: 10.1038/s41598-024-60467-y (PMC11061309; doi:10.1038/s41598-024-60467-y)
Supplement: Supplementary file 1 — Supplementary Information. [file 41598_2024_60467_MOESM1_ESM.docx]

**Supplementary Information for**

**Role of volcanism and impact heating in mass extinction climate shifts**

Kunio Kaiho

Email: kunio.kaiho.a6@tohoku.ac.jp

**This PDF file includes:**

Supplementary Table 1

Supplementary Table 1. Coronene index values during heating events in mass extinctions

| Age | Section | Country | Sample | Coronene |
| --- | --- | --- | --- | --- |
|  |  |  |  | Index |
| K–Pg boundary | Caravaca | Spain | SPCA1～2 | 0.90 |
| K–Pg boundary | Caravaca | Spain | SPCA+0.5～+1 | 0.89 |
| K–Pg boundary | Caravaca | Spain | SPCA+0.2～+0.5 | 0.88 |
| K–Pg boundary | Caravaca | Spain | SPCA0～+0.2 | 0.88 |
| K–Pg boundary | Beloc | Haiti | HIST72～76 | 0.88 |
| K–Pg boundary | Beloc | Haiti | HIST65～72 | 0.93 |
| K–Pg boundary | Beloc | Haiti | HIST55～60 | 0.89 |
| K–Pg boundary | Beloc | Haiti | HIST50～55 | 0.92 |
| K–Pg boundary | Beloc | Haiti | HIST40～45 | 0.82 |
| K–Pg boundary | Beloc | Haiti | HIST30～35 | 0.85 |
| K–Pg boundary | Beloc | Haiti | HIST25～30 | 0.84 |
| End-Triassic | Kuhjoch | Austria | ASKJ21 | 0.10 |
| End-Triassic | Kuhjoch | Austria | ASKJ20 | 0.10 |
| End-Triassic | Kuhjoch | Austria | ASKJ19 | 0.07 |
| End-Triassic | Kuhjoch | Austria | ASKJ18 | 0.07 |
| End-Triassic | Kuhjoch | Austria | ASKJ17 | 0.05 |
| End-Triassic | Kuhjoch | Austria | ASKJ16 | 0.04 |
| End-Triassic | S Audrie's Bay | UK | UKSAB60-62 | 0.09 |
| End-Triassic | S Audrie's Bay | UK | UKSAB58-60 | 0.08 |
| End-Triassic | S Audrie's Bay | UK | UKSAB55-58 | 0.18 |
| End-Triassic | S Audrie's Bay | UK | UKSAB52-55 | 0.10 |
| End-Triassic | S Audrie's Bay | UK | UKSAB49-52 | 0.08 |
| End-Triassic | S Audrie's Bay | UK | UKSAB46-49 | 0.09 |
| End-Triassic | S Audrie's Bay | UK | UKSAB44-46 | 0.10 |
| End-Triassic | S Audrie's Bay | UK | UKSAB41-44 | 0.09 |
| End-Triassic | S Audrie's Bay | UK | UKSAB40-41 | 0.11 |
| End-Triassic | S Audrie's Bay | UK | UKSAB37-40 | 0.13 |
| End-Triassic | S Audrie's Bay | UK | UKSAB34-37 | 0.11 |
| End-Triassic | S Audrie's Bay | UK | UKSAB32-34 | 0.09 |
| End-Triassic | S Audrie's Bay | UK | UKSAB28-31 | 0.12 |
| End-Triassic | S Audrie's Bay | UK | UKSAB26-28 | 0.11 |
| End-Triassic | S Audrie's Bay | UK | UKSAB24-26 | 0.11 |
| End-Triassic | S Audrie's Bay | UK | UKSAB20-22 | 0.10 |
| End-Triassic | S Audrie's Bay | UK | UKSAB16.6-20 | 0.11 |
| End-Triassic | S Audrie's Bay | UK | UKSAB13.3-16.6 | 0.13 |
| End-Triassic | S Audrie's Bay | UK | UKSAB10-13.3 | 0.18 |
| End-Triassic | S Audrie's Bay | UK | UKSAB7-10 | 0.12 |
| End-Triassic | S Audrie's Bay | UK | UKSAB5-7 | 0.13 |
| End-Triassic | S Audrie's Bay | UK | UKSAB3-5 | 0.14 |
| End-Triassic | S Audrie's Bay | UK | UKSAB1-3 | 0.10 |
| End-Triassic | S Audrie's Bay | UK | UKSAB0-1 | 0.12 |
| End-Permian | Liangfengya | China | LFY 20 | 0.57 |
| End-Permian | Liangfengya | China | LFY – 19 (4-6) | 0.69 |
| End-Permian | Liangfengya | China | LFY – 19 (2-4) | 0.70 |
| End-Permian | Liangfengya | China | LFY – 19 (0-2) | 0.70 |
| End-Permian | Liangfengya | China | LFY 18 | 0.68 |
| End-Permian | Meishan | China | CHMI -1 – 0 | 0.51 |
| End-Permian | Bulla | Italy | BLA 8 (24-27 cm) | 0.45 |
| End-Permian | Bulla | Italy | BLA 8 (21-24 cm) | 0.43 |
| G–L boundary | Penglaitan | China | CHPLN0c | 0.72 |
| G–L boundary | Penglaitan | China | CHPLN0b | 0.89 |
| G–L boundary | Penglaitan | China | CHPLN0 | 0.44 |
| G–L boundary | Penglaitan | China | CHPL-1 | 0.77 |
| G–L boundary | Penglaitan | China | CHPL-2 | 0.82 |
| G–L boundary | Penglaitan | China | CHPL-5 | 0.83 |
| G–L boundary | Penglaitan | China | CHPL-7 | 0.75 |
| F–F boundary | Coumiac | France | FRCM31g4 | 0.89 |
| F–F boundary | Coumiac | France | FRCM31g3 | 0.89 |
| F–F boundary | Coumiac | France | FRCM31g2 | 0.88 |
| F–F boundary | Coumiac | France | FRCM31g1 | 0.89 |
| F–F boundary | Coumiac | France | FRCM31f | 0.88 |
| F–F boundary | Sinsin | Belgium | sin2~6 | 0.86 |
| F–F boundary | Sinsin | Belgium | sin0~2 | 0.87 |
| F–F boundary | Sinsin | Belgium | sin-2~0 | 0.86 |
| F–F boundary | Sinsin | Belgium | sin-6~-2 | 0.86 |
| F–F boundary | Sinsin | Belgium | sin-10~-6 | 0.95 |
| F–F boundary | Sinsin | Belgium | sin-14~-10 | 1.00 |
| F–F boundary | Sinsin | Belgium | sin-19~-14 | 0.96 |
| F–F boundary | Sinsin | Belgium | sin-27~-19 | 0.93 |

Data from Kaiho et al. ^1–5^.

**References**

1. Kaiho, K., Aftabuzzaman, M., Jones, D. S. & Tian, L. Pulsed volcanic combustion events coincident with the end-Permian terrestrial disturbance and the following global crisis. *Geology* **49**, 289−293 (2021a).

2. Kaiho, K. et al. Coronene, mercury, and biomarker data support a link between extinction magnitude and volcanic intensity in the Late Devonian. *Glob. Planet. Chang.* **199**, 103452 (2021b).

3. Kaiho, K. et al. Volcanic temperature changes modulated volatile release and climate fluctuations at the end-Triassic mass extinction. *Earth Planet. Sci. Lett.* **579**, 117364 (2022).

4. Kaiho, K., Grasby, S. E. & Chen, Z-Q. High-temperature combustion event spanning the Guadalupian−Lopingian boundary terminated by soil erosion. *Palaeogeogr., Plaeoclimatol., Palaeoecol.* **618**, 111518 (2023).

5. Kaiho, K. et al. Global climate change driven by soot at the K-Pg boundary as the cause of the mass extinction. *Sci. Rep*. **6**, 28427 (2016).
